# Charting Practices to Protect Against Malpractice: Case Reviews and Learning Points

**DOI:** 10.5811/westjem.2022.1.53894

**Published:** 2022-04-28

**Authors:** Summer Ghaith, Gregory P. Moore, Kristina M. Colbenson, Rachel A. Lindor

**Affiliations:** * Mayo Clinic Alix School of Medicine, Phoenix, Arizona; † Mayo Clinic, Department of Emergency Medicine, Phoenix, Arizona; ‡ Mayo Clinic, Department of Emergency Medicine, Rochester, Minnesota

## Abstract

**Introduction:**

Medical documentation issues play a role in 10–20% of medical malpractice lawsuits. Inaccurate, incomplete, or generic records undermine a physician’s defense and make a plaintiff’s lawyer more likely to take on a case. Despite the frequency of documentation errors in malpractice suits, physicians receive very little education or feedback on their documentation. Our objective in this case series was to evaluate malpractice cases related to documentation to help improve physicians’ documentation and minimize their liability risks.

**Methods:**

We used Thomson Reuters Westlaw legal database to identify malpractice cases related to documentation. Common issues related to documentation and themes in the cases were identified and highlighted.

**Results:**

We classified cases into the following categories: incomplete documentation; inaccurate text; transcription errors; judgmental language; and alteration of documentation. By evaluating real cases, physicians can better understand common errors of other practitioners and avoid these in their own practice.

**Conclusion:**

Emergency physicians can reduce their liability risks by relying less on forms and templates and making a habit of documenting discussions with the patients, recording others’ involvement in patient care (chaperones, consultants, trainees, etc.), addressing others’ notes (triage staff, nurses, residents, etc.), paying attention to accuracy of transcribed or dictated information, avoiding judgmental language, and refraining from altering patient charts.

## INTRODUCTION

More than 75% of emergency physicians will be named in a malpractice lawsuit at least once throughout their careers.^1^ Documentation issues are thought to play a role in up to 20% of these lawsuits.^2^ Previous studies of malpractice claims involving documentation indicate that these cases most commonly revolve around missing documentation (70%), inaccurate content (22%), or poor mechanics (18%).^3^ Poor mechanics includes errors in transcribed order, illegible entries, and delays in documentation.^3^ Physicians often focus on documentation as a means of communicating with other physicians and billing for their services, but it is also crucial to communicate with the patient and provide a legal record of the care provided. Often, malpractice lawyers decide whether to pursue litigation cases based solely on the quality of documentation. In malpractice cases, inaccurate, incomplete, or careless records undermine a physician’s defense and make a plaintiff’s lawyer more likely to take on a case.^2^

Despite the frequency of documentation issues in malpractice suits, physicians receive very little education on this topic through training and very little feedback on their documentation once in practice. The Accreditation Council for Graduate Medical Education does not specifically address or require medicolegal education, lending to varying levels of exposure and training on these topics. When surveyed, residents and physicians across multiple specialties reported receiving no medicolegal training at all, let alone training that is specific to documentation, and rated their knowledge as poor.^4^,^5^,^6^ Emergency physicians are particularly at high risk of documentation malpractice liability due to the large number of high-risk patients and fast-paced environment. The objective of this case series was to evaluate malpractice cases related to documentation errors and practices to help improve physicians’ documentation and minimize their liability risks. By evaluating real cases, physicians can better understand practices and common errors of other practitioners and avoid these errors in their own practice.

## METHODS

We used Thomson Reuters Westlaw, an online legal database, to search for medical malpractice cases related to documentation. Cases were classified into the following categories: missing documentation; inaccurate text; transcription errors; judgmental language; and alteration of documentation. Illustrative examples are provided below.

## RESULTS AND DISCUSSION

### Missing Documentation

Cases that involve missing documentation comprise a broad range of clinical circumstances.^2^ Common scenarios identified included lack of documentation about informed consent discussions, patients acting against medical advice (AMA), specialist consultations, and communication with patients regarding return precautions or post-discharge care. The cases below highlight situations in which missing documentation contributed to the physicians’ liability risks.

### Informed Consent

Physicians generally recognize the need to obtain informed consent and the risks of failing to do so, yet cases revolving around this issue remain common. Often in these cases, a standardized informed consent form is used, but the documentation is still deemed inadequate by the courts. For example, in *Brown v St. Clair Anesthesia, Ltd.*, a patient provided written consent for placement of a central venous catheter during a bypass procedure.^7^ However, the physician determined during the procedure that the patient needed a Swan-Ganz catheter instead. Unfortunately, when insertion was attempted, he perforated the vein and the patient died. The patient’s family alleged that the two types of catheters were different enough to warrant a separate and specific consent form. The hospital settled privately, and the physician took the case to court believing the initial form would protect him; he was found responsible for $1 million in damages.

In *Alaimo, Estate of v Berman*, a woman underwent a cosmetic breast surgery and developed a complication that was listed clearly on the informed consent form she had signed prior to the procedure.^8^ The patient argued that she was given the informed consent form just minutes before being wheeled to the operating room, and, therefore, her consent was rushed and not valid. Although the physicians involved in the case argued that she had been given the form much earlier, there was no timestamp on the actual form, and the court ruled in favor of the patient for a $3.5 million award. Although clinicians often assume that a signed, informed consent form protects them from procedural complications, especially those explicitly listed on the forms, these cases suggest that the forms’ protections are limited, and courts may expect more of a detailed conversation than a standardized form conveys.

### Patient Acting Against Medical Advice

While patients who leave AMA are widely recognized to be high risk when it comes to liability, we identified several cases in which physicians’ documentation of the encounter failed to protect them from liability. For example, in *McHone v Swedish Covenant Hospital*, emergency physicians recommended that a child with abdominal pain be transferred to a pediatric center for additional diagnostic studies.^9^ However, the mother wanted to drive the child herself. She signed an AMA form prior to discharge and was given instructions to present at the nearby children’s hospital. Rather than going right to the hospital, the mother stopped at her house, delaying her arrival at the referral center for several hours, and the child died due to sepsis from a ruptured appendix. Although the AMA form was signed and documented, the accompanying discussion was not. Other than the signed form, there was no evidence that the original physician really tried to ensure the mother understood the risks to her child or worked with her to come up with a safer plan, and the court found the physician partially liable for the child’s death.

Similarly, in *Parker v FL Emergency Physicians*, a patient arrived at the emergency department (ED) complaining of a headache concerning for a subarachnoid hemorrhage.^10^ Before the workup was completed, the patient left AMA and signed a form documenting this decision, his awareness of the risks, and his acceptance of those risks. Several days later, he died due to a ruptured aneurysm. Again, the AMA form itself did not convince the court that the physician adequately conveyed the risks to the patient or put sufficient effort into convincing him to stay, and the court issued a $9 million verdict. Finally, in *Tracy v Freund*, a patient went to the hospital with chest pain but chose to leave before his evaluation was complete.^11^ The patient signed an AMA form and had a fatal myocardial infarction a week later. This case occurred in a state in which the jury could apportion comparative fault for the parties involved. Comparative fault allocates negligence when both parties are at least somewhat at fault. They decided that the AMA form lessened the physician’s role but did not absolve him entirely, and they ultimately found him to be responsible for 50% of the damages, or $2.7 million.

These cases demonstrate that a signed AMA form is not sufficient protection from future liability and physicians should ensure that appropriate attention is directed toward this class of high-risk patients, both in encouraging them to stay and in fully documenting any efforts to convince them to do so. Additionally, physicians should document an assessment of a patient’s decision-making capacity in every AMA case, ideally with reference to the four elements of capacity, understanding, appreciation, reasoning, and communication.

### Discussions with Consultants

Consultations originating in the ED are another source of potential liability if not documented appropriately. In an anonymous case in New York, a patient presented to the ED with a headache.^12^ After an initial workup, the resident physician reported consulting a neurologist who recommended against additional diagnostic studies. The patient subsequently suffered a brainstem herniation from an undiagnosed subarachnoid hemorrhage and died in the ED. When the patient’s family brought suit, the resident defended himself by saying he was following the neurologist’s advice. The neurologist denied any recollection of the conversation, and there was no documentation to support that it had occurred. The jury felt that either the resident or the neurologist was being dishonest and awarded the patient’s family $44 million in damages.

Cases like this can be avoided by following a clear pathway for formal consultations, in which the consultant’s name is recorded, along with the time and content of the discussion, and consultants are made aware that their recommendations will be relied on and incorporated into the medical record. This does not preclude informal consultations, or “curbside consults,” in which a specialist’s advice is sought in an off-the-record fashion. In fact, instituting a clear pathway for formal consultations and being upfront about intention to document recommendations may alleviate consultants’ fears of being unknowingly named in the medical record and increase their willingness to provide informal input.

### Communicating with Patients at and After Discharge

While physicians’ documentation efforts tend to focus on the content of the clinical encounter, communication with patients at the time of and after discharge is equally important for minimizing liability. Lawsuits related to this issue may involve unclear referrals, inaccurate discharge instructions, incomplete return precautions, or failure to follow up on outstanding testing. For example, in *Hooten v Pediatrix Medical Group*, a newborn baby with retinopathy of prematurity was discharged after a prolonged hospital stay and referred to a local ophthalmologist for close follow-up.^13^ When the mother tried to follow up, she was told that physician was no longer available, and she was scheduled for an appointment a month later at a different practice. By the time she followed up, her child was blind. She argued that no one had told her about the importance of timely follow-up, and there was no documentation contradicting this, leading the court to issue a $9.25 million judgment in her favor.

In *Estate of Kimble*, poorly documented discharge instructions resulted in liability for a primary care physician.^14^ In this case, a woman presented to an outpatient clinic with shortness of breath and an elevated D-dimer. The physician instructed her to go to the ED and assumed the patient would do so but did not document this recommendation. When the patient instead went home and died from a massive pulmonary embolism, the physician settled for $2 million since she could not provide any evidence that there had been referral to the ED. Taking the time to discuss and document recommendations for post-discharge care minimizes physicians’ risks from these types of lawsuits.

Finally, test results that change or return after ED discharge can create liability risks for emergency physicians. Common scenarios requiring post-discharge follow-up include radiology reports that are later revised or tests that result after discharge, such as blood cultures or urine culture susceptibilities. The ED must have a process to provide these results to patients in a timely fashion, and emergency physicians should understand that they maintain ultimate responsibility for the efficacy of these processes. In scenarios in which attempts to contact patients are unsuccessful, all attempts to do so should be thoroughly documented. Maintaining an awareness of these processes and potential pitfalls can reduce physicians’ liability risks for discharged patients.

### Inaccurate Documentation

Separate from the issue of missing documentation, inaccurate documentation makes up the second most common category of documentation-related malpractice cases. Common issues in this category include using inaccurate templates, copying and pasting from other notes, and providing information that conflicts with other clinicians for the same encounter. Each of these issues has become more problematic with the shift to electronic health records (EHR).

Reliance on templates that automatically populate a normal physical exam or review of systems is a commonly used but risky practice. Examples of this include a review-of-systems template that records “no chest pain” for a patient with a chief complaint of chest pain, or a templated physical exam saying “moves all 4 extremities” when a patient has an amputation. Even if these mistakes have no impact the outcome of a patient’s care, they can be used to discredit the physician by persuading a jury that the physician was careless, rushed, and ultimately negligent in their care of the patient, based simply on one obvious mistake like this.

Inaccuracies in documentation also arise when physicians’ notes conflict with those of other healthcare personnel involved in the same encounter, such as triage nurses, non-physician staff such as physician assistants or advanced practice providers, or trainees. For example, in *Plaintiff v Defendant*, a patient presented to the ED with right-sided chest pain, had an unremarkable subsequent evaluation in the ED, and was admitted for pain control.^15^ In the hospital, he was eventually diagnosed with a spontaneous chest wall hemorrhage but unfortunately died of hemorrhagic shock. The family sued the physicians involved for not recognizing the acuity of the patient’s condition earlier. The emergency physician argued that the patient did not appear ill while in the ED, and referred to his own documented physical exam, which was normal. However, the patient’s family highlighted the nurse’s triage note, which described the patient as “cool, moist, and mottled” at arrival. They used this to argue that the physician’s exam was inaccurate and that the patient had shown signs of shock on arrival. The court agreed with the family, and they were awarded $800,000.

Similarly, in *Prager v Campbell Memorial Hospital*, a patient presented after involvement in a motor vehicle collision, and the triage nurse noted his chief complaint as neck pain.^16^ The physician’s chart indicated the patient complained only of upper back pain with a normal neck exam, and he discharged the patient after the imaging of his head and thoracic spine was reassuring. The patient woke up the next day with paralysis of one arm, was re-evaluated, and found to have an unstable cervical spine fracture, resulting in permanent arm weakness. The court ruled in favor of the patient for a $9 million verdict, based on the nurse’s note that documented the presence of neck pain at his initial visit.

The use of other healthcare professionals’ notes to cast doubt on the accuracy of physicians’ evaluations, as illustrated above, is actually a frequent strategy for lawyers. In comparing a physician note to a nurse’s note, trial lawyers teach, “the value of recognizing the difference between a brief note of a busy physician and the more time allowing leisurely and more explicit account of a nurse in a closer and more exposed encounter with a given patient. Cast in the proper light, the nurse’s notes may well be given more credence by a jury when confronted with a conflict reflecting significantly on either the client’s injury or the question of liability.”^2^ This observation highlights the need to be aware of what others have written about your patients and be proactive about addressing any inconsistencies.^2^ This risk is magnified for physicians overseeing non-physician personnel or trainees and highlights the risks of the common practice of signing off on charts after patient discharge or without full review.

### Transcription Errors

Transcription errors are a major source of liability for physicians and have become increasingly common with the shift to EHRs. The most common transcription errors include enunciation errors (53.9%), deletions (18.0%), and insertions (11.7%).^17^ Studies have found an average of seven errors per 100 words in electronic records, and a clinically significant error every 250 words.^17^ In the ED, 15% of notes have a clinically significant transcription error.^17^

Enunciation errors generally involve transcription or dictation systems misinterpreting spoken orders. For example, in *Juno v Amare*, insulin dosing provided in a patient’s discharge summary was transcribed by an outside transcription service as 80 units rather than 8 units, leading to the death of a patient.^18^ Despite the obvious technical fault in this case, rather than any impairment or deliberate negligence on the part of the physician, the court awarded the patient’s family $140 million. In *Madigan v Makavana*, a hospitalized patient with a known seizure history was receiving 150 milligrams (mg) of Keppra rather than 1500 mg due to a similar error, resulting in a seizure that caused a permanent neurologic deficit.^19^ The court ruled in favor of the patient for an $11.2 million verdict. The routine use of facial coverings in healthcare settings has the potential to significantly exacerbate these enunciation issues.

The use of EHRs also increases the risk of other types of errors, such as placing orders for the wrong patient or choosing the wrong options from a drop-down menu. In *Estate*, for example, a 91-year-old man who was in the ED for a mechanical fall was given high-dose chlorpromazine meant for a different patient.^20^ This error caused the patient’s death and resulted in a $750,000 settlement. In *Walrath v Smith*, a patient with hypokalemia was given discharge instructions for hyperkalemia.^21^ Despite verbal instructions to increase her potassium supplement, she followed her discharge instructions, decreased her potassium supplement, and arrested at home, resulting in a $100,000 settlement. Despite the role of technology in these cases, the physicians involved can be held accountable in the same way as if they had missed a diagnosis or chosen the wrong treatment.

### Judgmental Language

Use of judgmental language represents another potential documentation pitfall for physicians. In *Young v. Women’s Health*, a physician documented that a patient had a history of substance abuse, despite her denying this and providing proof for her claims.^22^ However, the information was left in the chart, and the patient was denied life insurance coverage based on this information. She was able to demonstrate that the information was false, and the courts sided with the patient for a verdict of $1.5 million. Judgmental language also comes in the form of providing unnecessary quotations that highlight the vernacular of a patient or clinically irrelevant details. For example, a chart that quotes a patient as reporting she has “the sugars” is unnecessary, creates a mocking tone, and will make it easy for an attorney to paint a picture that a physician feels superior to the patient. Similarly, a chart that alludes to a patient’s appearance, religion, or political party, if not otherwise relevant, can easily be used by a trial attorney to suggest the physician was biased against the patient. In addition, studies have shown that physicians’ use of negative details and quotations in patients’ charts tends to negatively bias downstream clinicians.^23^, ^24^ Keeping unnecessary details and quotations out of the record can shield physicians from this this type of claim and protect patients from unnecessary bias.

### Alterations in Charting

Another common issue is the alteration of previously recorded documentation. In *Perry v United States*, a five-week-old patient was brought to the ED twice in the same day with a fever, was seen by the same physician and discharged without appropriate testing.^25^ On the third visit, the patient was diagnosed with meningitis and suffered a permanent neurologic deficit. The physician altered the charts from previous visits to obscure the fact that the patient had a fever, but this was easily identified in court proceedings, and the court levied a $20 million verdict against him.

In *Lei*, a 21-month-old patient died after a delayed diagnosis of an incarcerated hernia.^26^ While the delay in diagnosis may have been reasonable, the documentation was changed prior to trial to delete a note about the patient’s “bilious vomiting,” contributing to a $3.28 million verdict in favor of the patient. Lastly, in *Buchanan v Metrolina Medical Associates*, a patient presented to the ED with shortness of breath and chest pressure that originated during prolonged travel.^27^ The physician ordered a chest radiograph, which was negative, and discharged the patient on an antibiotic. The patient died the next day due to a pulmonary embolus. During the trial, the metadata was used to prove that the physician went into the patient’s chart after his death to indicate that the patient had declined an electrocardiogram, that the cough was productive, and that a calf exam had been performed. While these notes may have been true, their entry after the fact raised the specter of a cover-up and forced the physician into a $3 million settlement.

Documentation alteration is relatively easy to identify because EHRs contain meta-data that can demonstrate timestamps for nearly every change and review of a page in the record. The best way to avoid this situation is to document fully at the initial patient encounter; however, if it is necessary to go into a chart and document at a later date, especially in patients with a known bad outcome, physicians should acknowledge that they are doing so by documenting the date and why the changes are being made to the chart. While this may still lead to some loss of credibility by the readers, it is the only way to addend a patient’s chart without casting doubt on one’s intentions.

Besides looking dishonest, alteration of documentation can have several other consequences. For example, many states can revoke physicians’ licensure if they are found to have altered a record. In addition, some malpractice insurance companies will not provide coverage for physicians if they altered records, leaving them vulnerable to the entirety of a verdict or settlement. Similarly, in some states where punitive damages have been banned or capped as a form of tort reform, these limits do not apply in cases of document alteration. Finally, in some courts, document alteration reverses the evidentiary burden, meaning that patients no longer have to prove that a physician harmed them, but rather the physicians have to prove that they did not. The myriad of consequences associated with alteration of documentation emphasizes the danger of this practice and the importance of documenting appropriately at the initial encounter.

## LIMITATIONS

The above content provides qualitative information designed to highlight potential areas of vulnerability for clinicians. Due to the nature of the database, it is not possible to provide a quantitative assessment of risk for each of the areas described. Similarly, the case examples provided may not be representative of the most common cases in each category. These limitations notwithstanding, we feel the examples included here provide valuable insight into several areas in which documentation issues can heighten physicians’ liability risks, guided by previous studies on this topic.

## CONCLUSION

Risk of malpractice cases involving documentation can be minimized by understanding common errors and practices that lead to lawsuits. These errors are relatively easy to commit; recognizing these potential pitfalls will not only decrease the likelihood of a malpractice lawsuit but also decrease the risk of contributing to an adverse patient outcome. Emergency physicians can reduce their liability risks by relying less on forms and templates and making a habit of documenting discussions with the patient, recording others’ involvement in patient care (a chaperone, consultant, trainee, etc.), addressing other caregivers’ notes (triage, nursing, residents, etc.), paying attention to accuracy of transcribed or dictated information, avoiding judgmental language, and refraining from altering patient charts. This case series is not meant to encourage physicians to document more but rather more effectively, highlighting specific parts of the chart that have historically been problematic and may warrant more attention.
